# Factors associated with 1-year outcomes and transient intraocular pressure elevation in minimally invasive glaucoma surgery using Kahook Dual Blades

**DOI:** 10.1038/s41598-023-42575-3

**Published:** 2023-09-14

**Authors:** Tomoaki Sakamoto, Hirokazu Nisiwaki

**Affiliations:** Department of Ophthalmology, Tenri Hospital, 200 Mishimacho, Tenri, Nara 632-8552 Japan

**Keywords:** Diseases, Medical research

## Abstract

In this retrospective case–control study, we aimed to investigate the mid- to long-term outcomes and factors involved in minimally invasive glaucoma surgery using the Kahook Dual Blade. Of the 229 cases since 2018 in which the dual blades were used for glaucoma surgery at the Tenri Hospital, 133 eyes of 98 patients who followed up for more than 3 months were included. Intraocular pressure (IOP), number of drops score, and need for reoperation were evaluated on day 1 and at 1, 3, 6, 9, and 12 months postoperatively. Intraocular pressure spikes occurred in 25 patients postoperatively (18.8%), occurring at approximately 4.5 days (1–10.25). The preoperative number of eye drops used and ocular axial length were found to be associated with the occurrence of spikes (OR = 1.45, 95% CI 1.02–2.06; P = 0.025 and OR = 1.41, 95% CI 0.98–1.25; P = 0.072, respectively). At the 12-month mark, no significant relationship was found between the presence of spikes or incisional extent scores and the amount of change in IOP and number of drops scores. Patients with severe visual field impairment, high preoperative IOP and drop scores, and long ocular axial length may require more frequent follow-ups after surgery to check for spikes.

## Introduction

Glaucoma is one of the leading causes of irreversible blindness, greatly affecting one’s quality of life and vision^1,2^. Significant intraocular pressure (IOP) reduction is important in glaucoma management to prevent optic nerve injury^3,4^. Methods to reduce IOP include use of eye drops, laser therapy, and surgery.

Generally, incisional surgeries are performed when other treatments are ineffective. Trabeculectomy and tube-shunt implantation can provide greater IOP reduction than other treatments^5^. However, these surgeries require high expertise and frequent postsurgical follow-up, with an increased risk of developing postoperative complications such as excessively low IOP and infection^6–8^.

Trabeculotomy is a method of incising the trabecular meshwork ™ and lowering the aqueous humor outflow resistance^9–11^. However, conventional ab externo trabeculotomy is invasive, requiring conjunctival and scleral incisions and suturing^12^.

Minimally invasive glaucoma surgery (MIGS), a recently developed procedure, has been found to be a faster and more effective technique in moderate glaucoma treatment^13–15^. MIGS has gained popularity because of its favorable safety profile, ability to be combined with cataract surgery, and the reduction or elimination of adjunctive glaucoma medications. MIGS is particularly effective in reducing the number of eye drops required in patients with poor adherence to eye drop therapy and who develop adverse eye drop reactions.

The Kahook Dual Blades (KDB; New World Medical, Rancho Cucamonga, CA, USA) is an ab interno trabeculotomy that enables the removal of the TM and inner walls of Schlemm’s canal using dual blades to decrease resistance to aqueous outflow^16–23^. Preclinical studies have shown that near total resection of the TM without scleral damage can be achieved^17^. Numerous studies have also indicated that the KDB effectively lowers IOP and reduces the number of eye drops required. Recently, favorable long-term results have been reported at the 3-year mark^23^. At 12 months, the success rate was 71.8% for the phacoemulsification-KDB group and 68.8% for the KDB-alone group^24^. Goniotomy with the KDB lowered IOP significantly more than iStent implantation, with few adverse events in both groups^25^.

The aim of this study was to examine factors related to the treatment effects and complications in KDB over a medium-to-long-term period with a large sample size.

## Methods

This retrospective, case–control study was approved by the institutional review board of the Tenri Hospital (Approval no: 1312), which waived the requirement for informed consent owing to the retrospective nature of the study and the use of anonymized data. All methods were carried out following relevant guidelines and regulations. The study design, target patients, data details to be used, and principal investigators are posted on the hospital’s official website. Patients who refused to participate in the study were removed from the data set. The study adhered to the tenets of the Helsinki Declaration.

### Patient selection and measured variables

Of the 229 cases who underwent minimally invasive glaucoma surgery using KDB since its introduction at our hospital in 2018, we included 133 eyes of 98 patients aged ≥ 18 years with no history of endophthalmic surgery except cataract surgery (procedures such as laser iridectomy were also excluded) and who could be followed for at least 3 months postsurgically.

Baseline variables included age, sex, visual acuity, preoperative IOP, preoperative eye drops scores, incision range, mean deviation or mean defect (MD) value of the visual field test, presence of errors in the visual field test for poor fixation, disease type, and ocular axis length. The best-corrected visual acuity immediately before surgery was used, which was converted to the logarithm of the minimum angle of resolution (logMAR) visual acuity for statistical analysis. The indications for surgery include cases in which target IOP could not be achieved with eye drops, and those who cannot tolerate said topical therapy due to allergies, decreased cognitive function, et al. Preoperative IOP was recorded as the average of the IOP measured twice with a Goldmann applanation tonometer using the same eye drops as before KDB.

To identify the type of glaucoma, a gonioscopy lens was used to measure the patient`s anterior chamber angle. The drop score was defined as one and two points for one component and a fixed-dose combination, respectively. Oral IOP-lowering medications were considered as one component and scored as one point.

Incisional range was determined from surgical records and divided into three quadrants: range 1 for cases narrower than the first quadrant, range 2 for cases between the first and second quadrants, range 3 for cases between the second and third quadrants, and range 4 for cases wider than the third quadrant.

For visual field testing, we used the MD value of the Humphrey visual field test immediately before surgery. An MD greater than − 6 dB was classified as mild; if less than − 6 dB or greater than − 12 dB, moderate; and less than − 12 dB, severe.

The ocular axis length was measured using an optical coherence ocular axis length measuring device (IOLmaster 500; Carl Zeiss Meditec, Jena, Thuringia, Germany).

IOP and drop scores one day and 1, 3, 6, 9, and 12 months after surgery were recorded as postoperative measurement points. Postoperative complications included transient elevation of IOP (spike) and hyphema. Spike was defined as an increase in IOP of 10 mmHg or more from baseline within the first postoperative month. We used drops or oral/IV drug infusion when the event happened. Hyphema was considered significant if its size was greater than 1 mm.

The primary endpoint was to assess factors associated with a spike occurrence. Secondary endpoints included examining factors related to changes in IOP and eye drop use at 12 months and compared scores after categorizing patients according to preoperative IOP value (≤ 18 mmHg vs. > 18 mmHg). Survival analysis was also performed, with death defined as two consecutive IOP drops < 20% or need for additional glaucoma surgery.

### Surgical techniques

Patients were anesthetized using ophthalmic anesthesia (4% xylocaine), incisions were made with a microvitreoretinal knife at the 10 and ’2 o'clock positions, and space was secured at the corner angle with purified sodium hyaluronate/sodium chondroitin sulfate (Viscoat 0.5; Alcon Japan Ltd, Toranomon, Minato-ku, Tokyo). We used a 2.2-mm corneal incision and in-the-bag intraocular lens placement during cataract surgery with phacoemulsification.

The patient's head was tilted, and the corner angle assessed using a corner angle speculum; the KDB was inserted into the TM, and nasal, inferior, and auricular fibroblasts were removed. After checking for blood reflux, the anterior chamber was rinsed, and the wound was closed with intraocular fluid before completing the surgery.

During hospitalization, all postoperative patients were treated with 1.5% levofloxacin and 0.1% betamethasone valerate + gentamicin sulfate eye drops. After discharge from the hospital, the latter was changed to 0.1% fluorometholone and patients were advised to continue instillation for approximately 1 month. Bromfenac sodium hydrate was also used for patients simultaneously undergoing cataract surgery.

### Statistical analysis

For each variable, Mann–Whitney U and Fisher’s exact tests were performed to check for spike incidence, and logistic regression analysis was performed for preoperative eye exam variables. Wilcoxon signed-rank test was performed to analyze each examination point preoperatively.

Linear regression analysis was done to examine the factors involved in the change in IOP and drop score between preoperative and 12-month postoperative time points. For grading by the incisional extent and visual field testing, both IOP and ophthalmic scores at 12 months postoperatively were compared between the groups using the Kruskal–Wallis test. The survival rates were compared using the log-rank test for range 1 and 4 incisions. Post-hoc analysis (P value adjusted via the Holm method) was performed to examine the IOP and ocular score association between each examination point. Kaplan–Meier curves were drawn for the survival analysis. The last observation carried forward was used to complete the missing IOP values.

The P-values were two-tailed, and the significance level was set at 5%.

## Results

### Patient characteristics

Ultimately, 133 eyes of 98 patients were available for analysis: 74 eyes of 56 male patients and 59 eyes of 32 female patients. The age of the patients was 73.4 (mean) ± 8.18 (SE) years, visual acuity was 0.198 ± 0.267 in logMAR, preoperative IOP was 19.2 ± 5.16 mmHg, preoperative drop scores were 2.85 ± 1.5, and MD was − 11.05 ± 7.74. Twenty-five patients had poor fixation (Table 1).

**Table 1:** Patients’ baseline characteristics.

	Total	IOP ≤ 18 mmHg	IOP > 18 mmHg
Number of eyes	133	61	72
Age (y), mean (SE)	73.4 ± 7.96	74.6 ± 7.36	72.4 ± 8.41
Sex, n (%)			
Male	74 (56)	32 (52)	42 (58)
Female	59 (44)	29 (48)	30 (42)
IOP (mmHg), mean (SE)	19.2 ± 5.16	15.0 ± 2.09	22.7 ± 4.25
Drop score, n, mean (SE)	2.85 ± 1.5	2.70 ± 1.41	2.97 ± 1.57
BCVA (logMAR), mean (SE)	0.198 ± 0.267	0.147 ± 0.175	0.24 ± 0.32
MD (dB), mean (SE)	− 11.05 ± 7.74	− 11.4 ± 7.37	− 10.7 ± 8.08
Axial length (mm), mean (SE)	24.8 ± 1.84	24.7 ± 1.77	24.8 ± 1.92

*BCVA* best corrected visual acuity, *dB* decibel, *IOP* intraocular pressure, *logMAR* logarithm of the minimum angle of resolution, *MD* mean deviation, *mmHg* millimeters of mercury, *SE* standard error.

In addition, 116 patients underwent simultaneous cataract surgeries, while 17 underwent KDB procedures alone. The cases included: 95 primarily open-angle glaucoma, 14 normal-tension glaucoma, 12 pseudoexfoliation glaucoma, 10 ocular hypertension, and 2 steroidal glaucoma.

### Factors contributing to the occurrence of a spike

A spike was observed in 25 cases (18.8%). The timing at which spikes were observed was 4.5 days (1–10.25). Univariate analysis of spike incidence showed significant differences in the preoperative drop scores and ocular axial length (P = 0.002 and P = 0.02, respectively) (Table 2). Preoperative drop scores and ocular axis lengths were used as explanatory variables in the multivariate analysis. Logistic regression analysis similarly found significant associations between these two variables, with an odds ratio of 1.45 (95% CI 1.02–2.06; P = 0.025) for preoperative drop scores and 1.41 (0.98–1.25; P = 0.072) for ocular axis length.

**Table 2:** Comparing patients' baseline characteristics with occurrence of a postoperative spike.

Spike occurrence	Present	Absent	P-value
Number of eyes	25	108	
Sex, n (%)			
Male	17 (68)	57 (53)	0.19
Female	8 (32)	51 (47)	
Age (y), mean (SE)	70.4 ± 10	74.1 ± 7.33	0.1
Baseline IOP (mmHg), mean (SE)	19.5 ± 4.93	19.1 ± 5.23	0.63
Baseline drop score (n), mean (SE)	3.68 ± 1.44	2.66 ± 1.45	0.002
With cataract surgery, n (%)	23 (92)	92 (85)	0.62
Axial length (mm), mean (SE)	25.6 ± 2.23	24.5 ± 1.68	0.02
BCVA (logMAR), mean (SE)	0.23 ± 0.36	0.19 ± 0.24	0.83
Hyphema	2	8	1
MD classification			
Mild	5	35	0.39
Moderate	8	30	
Severe	13	42	
Incision range classification			
Range 1	7	25	0.31
Range 2	8	22	
Range 3	4	30	
Range 4	4	40	

*BCVA* best corrected visual acuity, *IOP* intraocular pressure, *logMAR* logarithm of the minimum angle of resolution, *SE* standard error.

### IOP and drop scores preoperatively and at 12 months postoperatively

**Figure 1:**
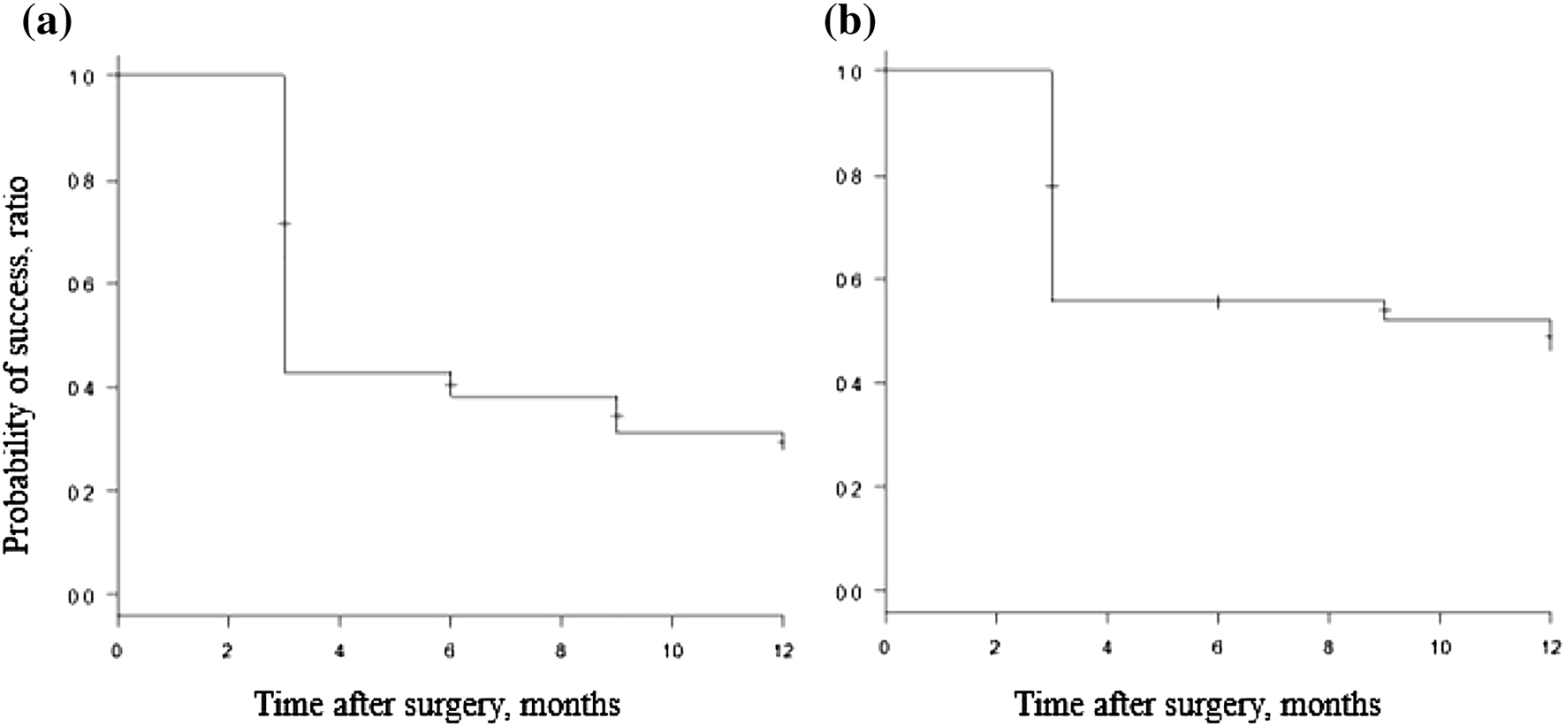
Kaplan–Meier survival curves using the previously described survival criteria. (**a**) Survival curves for all patients. (**b**) Survival curves for patients with preoperative IOP > 18 mmHg.

A significant difference was observed between the preoperative and 12-month postoperative IOP values (P < 0.001) (Table 3). The degree of IOP change was related to preoperative and postoperative Day 1 IOP (P < 0.001 and P = 0.01, respectively), and a significant difference in eye drop scores was observed between the preoperative period and 12 months postoperatively (P < 0.001). Linear regression analysis showed significant associations between age and preoperative eye drop score (P = 0.01 and P < 0.001, respectively) (Table 4). The survival rate was 27.8% at 12 months (Fig. 1). Seven patients needed additional glaucoma surgery.

**Table 3:** Result of IOP and drop scores at each postoperative measurement point compared with baseline.

	Baseline	Day 1	1st month	3rd month	6th month	9th month	12th month
Number of eyes	133	133	133	133	120	96	86
IOP (mmHg), mean (SE)	19.2 ± 5.16	17.0 ± 7.46	16.6 ± 4.56	15.7 ± 3.4	15.4 ± 2.78	15.5 ± 2.51	15.4 ± 3.22
P-value (IOP mean change from baseline)	–	< 0.001	< 0.001	< 0.001	< 0.001	< 0.001	< 0.001
Drop score, mean (SE)	2.85 ± 1.5	–	1.19 ± 1.4	1.67 ± 1.48	1.86 ± 1.59	1.91 ± 1.62	2.14 ± 1.74
P-value (drop score change from baseline)	–	–	< 0.001	< 0.001	< 0.001	< 0.001	< 0.001

*IOP* intraocular pressure, *SE* standard error.

**Table 4:** Univariate linear regression analysis for the degree of change in IOP and drop score.

Amount of change	IOP		Drop score	
	Coefficient (95% CI)	P-value	Coefficient (95% CI)	P-value
Age	− 0.06 (− 0.17–0.07)	0.38	− 0.06 (− 0.1–0.002)	0.01
Sex	− 1.87 (− 3.80–0.05)	0.06	− 0.62 (− 1.27–0.03)	0.06
With cataract surgery	0.7 (− 2.62–4.04)	0.68	− 1.1 (− 2.18–0.03)	0.06
BCVA (logMAR)	− 2.67 (− 6.28–0.94)	0.14	− 0.27 (− 1.45–0.91)	0.65
MD classification	− 0.96 (− 2.15–0.23)	0.11	− 0.04 (− 0.45–0.36)	0.83
Incision range classification	− 0.26 (− 0.62–1.14)	0.56	0.03 (− 0.33–0.27)	0.86
Day 1 IOP (mmHg)	− 0.16 (− 0.29–0.004)	0.01	− 0.02 (− 0.02–0.07)	0.26
Baseline IOP (mmHg)	− 0.73 (− 0.87–0.59)	< 0.001	0.06 (− 0.01–0.12)	0.1
Baseline drop score	− 0.1 (− 0.78–0.59)	0.78	− 0.33 (− 0.55–0.11)	< 0.001
Axial length (mm)	− 0.11 (− 0.43–0.65)	0.68	0.08 (− 0.1–0.26)	0.39
Spike	− 1.3 (− 3.54–0.94)	0.25	0.18 (− 0.67–1.13)	0.67

*BCVA* best corrected visual acuity, *CI* confidence interval, *IOP* intraocular pressure, *logMAR* logarithm of the minimum angle of resolution, *MD* mean deviation.

### IOP and drop score categorization

**Figure 2:**
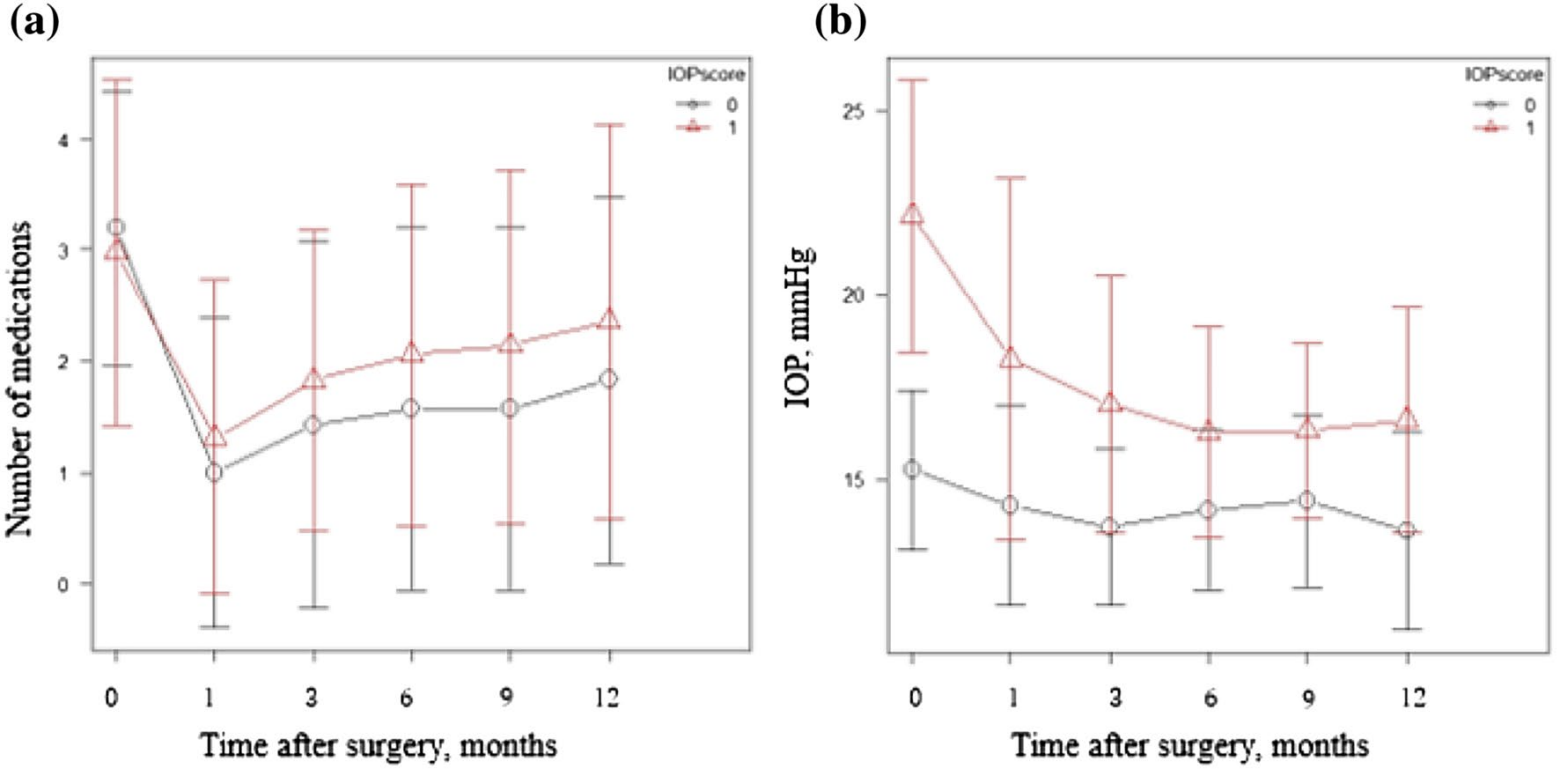
Changes in the number of medications and IOP over time. (**a**) Change in the number of medications. (**b**) Change in IOP value. 〇 indicates cases where the preoperative IOP was ≤ 18 mmHg; △ indicates cases where the preoperative IOP was > 18 mmHg. The error bar shows the standard deviation.

IOP and drop scores were categorized into cases with preoperative IOP ≤ 18 mmHg and those with IOP > 18 mmHg (Fig. 1). Post-hoc analysis showed significant differences in both changes in IOP and drop score (P < 0.001) (Fig. 2). Multiple comparisons showed significant differences in IOP between each measurement point compared to the preoperative IOP, but no significant differences between the different postoperative measurement points were noted. As for the drop score, a significant difference was observed between the postoperative and preoperative eye drop score. There was also a significant difference between the first postoperative month and subsequent measurement points (P < 0.001).

### Comparison of IOP and drop scores among groups classified by the extent of the incision

No significant difference was observed in IOP at 12 months postoperatively among the four groups classified by incision range (range 1: 60–100°, range 2: 100–120°, range 3: 120–130°, range 4: 130–260°; P = 0.36). The same was true for eye drop scores, with no significant difference observed among the four groups (P = 0.285). No significant difference was found in the survival analysis between the groups of ranges 1 and 4 (P = 0.44).

### Comparison of IOP and drop scores among groups classified by preoperative MD

No significant difference was found in survival rates among the three groups classified by preoperative MD (MD >  − 6.0 dB, mild; − 6.0 > MD > − 12.0 dB, moderate; and MD < − 12 dB, severe; P = 0.079). No significant difference was observed in drop scores and IOP at 12 months postoperatively (P = 0.8 and P = 0.09, respectively).

## Discussion

In this study, there was a significant association between preoperative eye drop score and ocular axial length with respect to the spike occurrence.

In patients preoperatively controlled with multiple medications, eye drops are expected to strongly inhibit aqueous humor production and enhance outflow. Generally, eye drops are discontinued postoperatively. The timing of the spike varies from case to case. Still, it is believed that the spike occurs when the outflow of aqueous humor is reduced for some reason after surgery, coinciding with the timing of the recovery of the individual's aqueous humor after surgery^26^. In addition, patients who are controlled with multiple medications may have a longer disease duration and show aqueous humor outflow tract atrophy after SC^27^. Notably, most patients with spikes are able to reduce their IOP with medication within 1–3 months; only a few patients require revision surgery due to persistent high IOP caused by spikes. It has been reported that ocular axis length also plays a role in the occurrence of spikes after cataract surgery^28^. There are also reports suggesting that high myopia may cause prolonged inflammation of the anterior chamber^29^, that the eye’s irregular shape may increase outflow resistance of the scleral collector channels^30^, that the depth of the anterior chamber may be so deep that viscoelastic material used during surgery may be left behind. Several reports mention the incidence of intraocular pressure spikes^22,25,31^. In the present study, the rate was 18.8%, similar to the 14% reported by Iwasaki et al. The spike incidence rate of Sieck et al., is 10%, which is slightly lower than our study. For this reason, Sieck et al. defined spike occurrence as happening 1 week postoperatively. In our study, the median time of spike occurrence was 4.5 days, and the third quartile was 10.25 days, indicating that spikes often occur after the first postoperative week. According to ElMallah MK et al., the incidence of spike is very low (1%), but no clear definition of spike is given. In summary, the 12-month postoperative outcome was not significantly related to the history of spike occurrence and may not significantly impact the surgical outcome. On the other hand, patients with high drop scores, visually end-stage, and potentially fatal spike IOP elevations may require frequent IOP checks during the first postoperative month. Since the multivariate analysis in the present study only suggested an association between IOP length and spikes, further studies are needed to increase the number of cases.

Linear regression analysis of the IOP change between the preoperative period and 12 months postoperatively revealed a significant association between the preoperative and postoperative day 1 in the amount of IOP change. Patients with high IOP preoperatively and on the first postoperative day may have a certain degree of IOP reduction at 12 months postoperatively. Linear regression analysis of the change in drop score between preoperative and 12 months postoperatively showed a significant involvement of age and preoperative drop score. Imbalances in response to age-related changes eventually cause an increase in fibronectin and the transformation of trabecular meshwork cells. Eventually, trabecular outflow and uveoscleral outflow are reduced^27^. Our analysis does not show an association between age and IOP changes. However, drop changes are associated with age. Even in elderly patients, the surgical outcome may not be adversely affected.

The incision range was grouped by quartile range and analyzed; no significant difference was found in each group's IOP and drop scores. A previous report using optical coherence tomography with Trabectome found no significant relationship between the incisional area and IOP reduction^32^. This is because aqueous humor flows through the TM, SC, and collector channels and then flows into the episcleral vein^11^. Although the TM and SC are located around nearly the entire angle of the corneal perimeter, there are only a limited number of collector channels, and even if the outflow resistance of the TM is reduced, the collector channels may develop a bottleneck situation, limiting the degree of IOP reduction. Therefore, even if the TM outflow resistance is lowered, the drop in IOP is limited. Thus, theoretically, a significant difference in IOP reduction may occur between a very small area and a 360° TM incision.

Many reports on 360° TM incisions have been conducted, some with favorable results from the viewpoint of complications^33,34^. In contrast, a report comparing the results of a 360-degree suture trabeculotomy incision with those of a KDB found no significant difference in IOP reduction^35^. Based on these results, the KDB may not require extremely large incisions, although it may be an option for extensive incisions only in cases where reoperation is difficult due to age or background.

The analysis classified according to preoperative MD values showed favorable results regardless of the degree of glaucoma progression, consistent with a previous report^21^. Outflow-tract surgery may also be indicated in cases of severe visual field defects. Another report found no significant difference between cataract surgery alone and combined KDB and cataract surgery in glaucoma patients with moderate-to-mild visual field impairment and good IOP control^18^. Cataract surgery alone may be acceptable in cases with good IOP control, except in cases where there is a strong objective to reduce the use of eye drops. In contrast, in cases with poor IOP control, the KDB may be recommended even in cases of moderate-to-severe visual field impairment. However, a spike may occur at a certain rate, and this risk should be fully explained to the patient.

This study’s limitation include the fact that it was a single-center study. Future studies should be conducted with a larger number of patients. Missing values were supplemented by the last observation carried forward. The incision range was treated as an ordinal variable using the interquartile range rather than a continuous variable because the surgeon's subjective findings based on the surgical records may lack reliability. Therefore, the quantitative evaluation of the incision range performed in this study is insufficient. Also, the observation period is short, and long-term follow-up studies are needed in the future.

## Conclusion

KDB may be effective in improving IOP and drop scores regardless of preoperative IOP, and the extent of TM incision and occurrence of a spike may have little effect on the postoperative course. Preoperative drop scores and ocular axial length are suggested to be involved in the risk of spike occurrence. Hence, patients with severe visual field impairment, high preoperative IOP and drop scores, and long ocular axial length may require more frequent follow-ups after surgery to check for spikes.

## Data Availability

The datasets generated during and/or analyzed during the current study are available from the corresponding author upon reasonable request.
